# The Reduction of Cervical Hyperlordosis and Resolution of Craniocervical Symptoms in an Adolescent Female: A Chiropractic Biophysics Case Report With Long-Term Follow-Up

**DOI:** 10.7759/cureus.69913

**Published:** 2024-09-22

**Authors:** Thomas J Woodham, Miles O Fortner, Jason W Haas, Paul A Oakley, Deed E Harrison

**Affiliations:** 1 Chiropractic Biophysics, Western Plains Chiropractic, Gillette, USA; 2 Research, CBP NonProfit, Inc., Windsor, USA; 3 Kinesiology and Health Science, York University, Toronto, CAN; 4 Chiropractic, Innovative Spine and Wellness, Newmarket, CAN; 5 Physical Medicine and Rehabilitation, CBP NonProfit, Inc., Eagle, USA

**Keywords:** chiropractic biophysics, neck pain, cephalgia, pediatric, migraine, cervical hyperlordosis, cervical lordosis

## Abstract

Cervical hyperlordosis is a rare condition in the pediatric population. We present a unique case of the application of Chiropractic Biophysics^®^ (CBP®) technique protocols to reduce a hyperlordotic cervical spine corresponding with many craniocervical symptoms, including chronic migraines and neck pain. A 15-year-old female presented with chronic headaches, neck pain, and neck stiffness among other complaints following a martial arts sprain injury several months prior. There were many positive orthopedic tests and limited range of motion. Radiographs revealed a cervical hyperlordosis and a right lateral head translation. CBP® treatment was given and involved cervical distraction traction as well as corrective exercises twice a week for 12 weeks, and then monthly for one year with a complementary home program. After 12 weeks, there was a full recovery from migraines and neck pain correlating with an 8° reduction in lordosis and correction of head translation. At 15 months, the patient remained well and achieved a 13° total reduction in the neck curve. This is the first case documenting the successful application of CBP® methods to reduce cervical spine hyperlordosis in peer-reviewed literature. We propose too much curve may be as detrimental as too little curve in the cervical spine with respect to causing adverse stresses and strains in the surrounding soft tissues leading to pathological processes and nociceptive tendencies.

## Introduction

Cervical lordosis develops in utero [1] and its lordotic shape is essential to protect the neural elements [2], optimize the distribution of loads throughout the cervical spine [3], foster normal vertebral coupling with head movement [4], and accommodate horizontal gaze [5]. Cervical spine biomechanical parameters, as measured from X-rays, are essential to evaluate normal and abnormal craniocervical relationships and are essential to determine optimal treatment [6].

Indeed, a normal lordotic cervical spine exists in adults [7-10] and is projected to be somewhere in the range of 20-42° [8-11], as measured by the posterior tangent method [12]. Importantly, the normal cervical spine for children and adolescents differs from that of adults. Kasai et al. presented data on pediatric cervical lordosis from the ages of two to 18 years for Cobb angles from segments C3-7 [13]. These Cobb values can be extended to include C2 by adding 2.7° [14] and also converted to posterior tangents by adding 9° [15]. A modified table of Kasai et al.'s [13] values converted to posterior tangents from C2 to C7 is presented by Harrison et al. [15] and Oakley and Harrison [16]. As far as we are aware, these values serve as the most complete data on the normative cervical lordosis in children/adolescents and should be used by clinicians who rehabilitate the cervical lordosis in pediatrics/adolescents.

It is well established that cervical lordosis can be increased in those with cervical hypolordosis by Chiropractic Biophysics^®^ (CBP®) technique methods [15,17-20]. In fact, a recent systematic review locating nine clinical trials found the average correction to range from 12° to 18°, after approximately 10-12 weeks and 30-36 treatments [21]. Another recent systematic review reported on the results from 41 located case reports/series where it was found that an average 14° improvement in cervical lordosis occurred after an average of 40 treatments over an average of 16 weeks [22]. Importantly, all the controlled trials and case reports/series document the relief of many craniocervical complaints. It seems common for symptomatic complaints to correlate with the reduction and loss of the cervical curve; however, on occasion, a patient may present with cervical hyperlordosis. The reduction of too much lordosis in the cervical spine is a rare occurrence in the literature; in fact, we could not locate such a case.

This case describes the unique application of the CBP^®^ technique to reduce a hyperlordotic cervical spine correlating with many craniocervical symptoms, including neck pain and headaches, in an adolescent.

## Case presentation

Patient history and initial symptoms

On December 27, 2021, a 15-year-old girl presented with her parents. She complained about neck pain with stiffness as well as headaches that began after martial arts-related activities approximately 7.5 months earlier. The parents noted that at one point, she had to be withdrawn from school for two weeks straight due to unbearable headaches. The patient reported many bodily complaints, including neck stiffness, middle neck pain, migraines up to six times per week with an aura of dissociation, jaw pain, ringing in the right ear, sinusitis, cold hands, cold feet, and middle back/shoulder blade pain. Table 1 represents the percentage improvement for all the symptoms after treatment.

**Table 1:** The patient's subjective improvement in initial symptoms at each re-examination and follow-up examinations.

Complaint	Exam 2	Exam 3	Exam 4	Exam 5
	7 weeks	12 weeks	9 months	15 months
Neck stiffness	20%	80%	90%	100%
Middle neck pain	70%	100%	100%	100%
Migraines (with aura)	10%	100%	100%	100%
Jaw pain	100%	100%	100%	100%
Ringing in the right ear	100%	100%	100%	100%
Sinusitis	50%	90%	90%	100%
Cold hands	80%	100%	90%	100%
Cold feet	20%	80%	90%	90%
Mid back/shoulder blade pain	20%	80%	90%	90%

Orthopedic test results and objective outcome measures

A visual posture exam revealed an obvious forward head posture. Strength testing showed weak left shoulder abduction with pain. The range of motion (ROM) of the cervical spine was restricted and painful on flexion, extension, and bilateral lateral bending. Deep tendon reflexes were normal. Sensory dermatome testing was unremarkable. Orthopedic tests were positive for bilateral cervical compression, Jackson’s compression, and maximal compression. Cervical distraction testing relieved the neck pain. Static palpation revealed hypertonicity, point tenderness, and motion restriction at C3, C4, T5, and L4. There were muscle spasms and/or trigger points of pain experienced by the patient in the cervical and trapezius areas at the time of testing. The neck pain disability index (NDI) [23] was scored and indicated a moderate disability (34%) and the headache disability index (HDI) [24] was scored and indicated a moderate disability (50/100).

Initial radiographic findings

**Figure 1:**
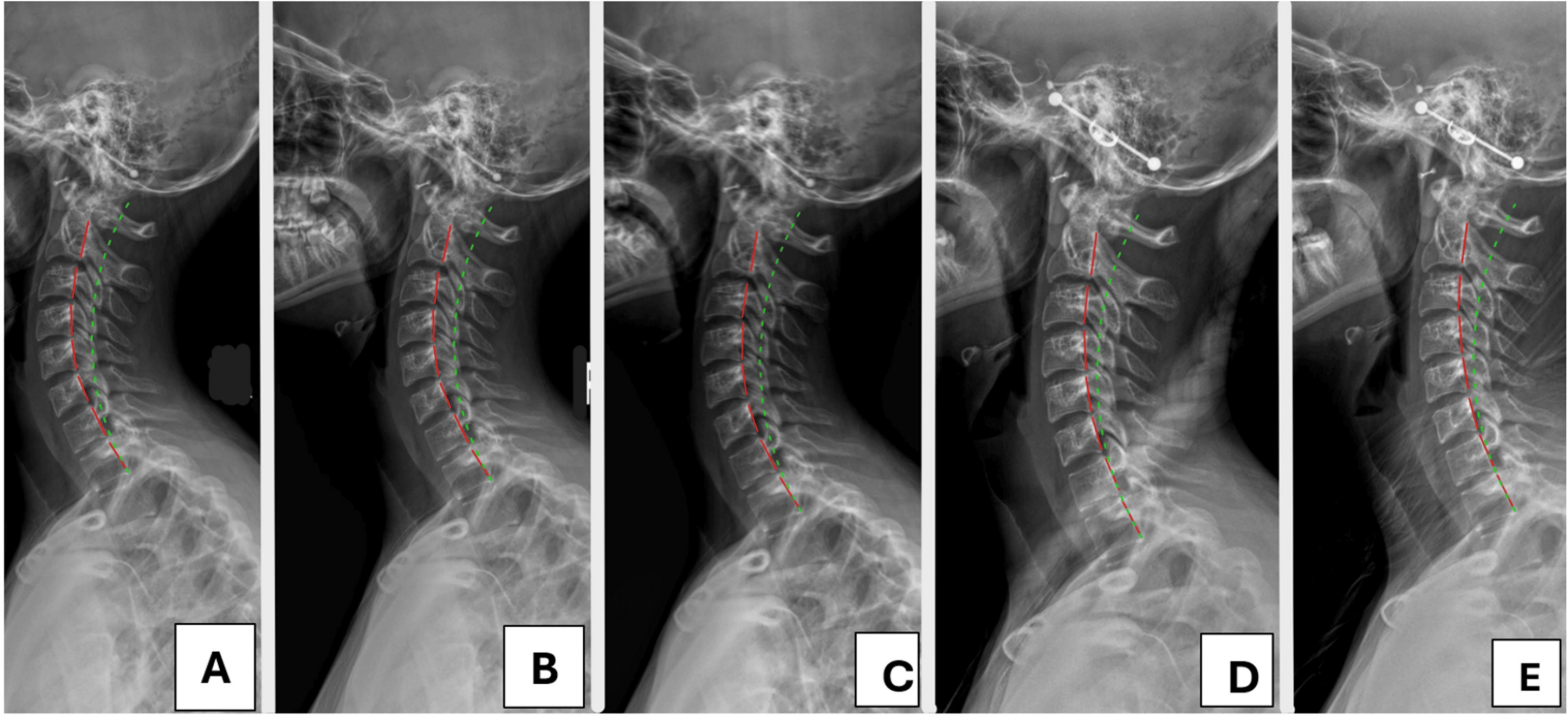
Lateral cervical radiographs from the initial (A) to the final follow-up examination (E). The green line represents the normal cervical lordosis, and the dashed red line represents the patient’s posterior vertebral lordosis contour. The patient progressed from significant hyperlordosis in panel A to a near normal lordosis following treatment and demonstrated stability with long-term follow-up in panel F.

**Figure 2:**
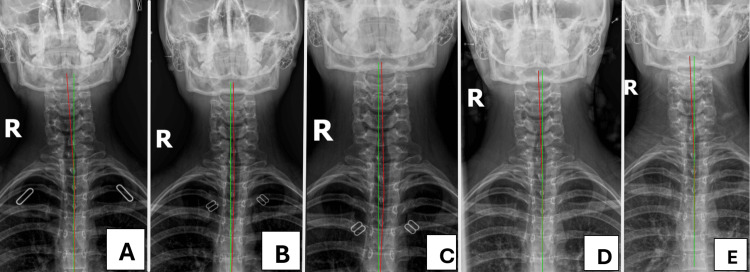
Anterior to posterior cervicothoracic radiographs from the initial examination (A) to follow-up examination (E). The patient’s right head translation improved following care and remained stable at long-term follow-up with only a slight return to baseline at panel E. The green line represents a vertical access line from the mid-thorax to the tip of the dens and the red line represents the best-fit line using the Risser-Ferguson analysis of the center of mass of the patient’s vertebral bodies.

Cervical radiographs were taken and digitized using the PostureRay® EMR (Trinity, FL, USA), a valid method [25] that uses the Harrison posterior tangent method (HPTM) to assess sagittal alignment (Figure 1) and the Risser-Ferguson method to assess coronal alignment (Figure 2) [12,26]. These methods are reliable and repeatable [12,26]. The lateral view showed hyperlordosis of the cervical spine (-44.0° vs. -25.4° adjusted normal) [16], increased atlas plane line (APL: -32.2° vs. 24-29° normal) [8,10], and a mild anterior head translation (AHT: 9.2 mm vs. 0 mm ideal). The anterior-posterior (AP) view showed a right head translation (8.5 mm vs. 0 mm ideal), a 4.7° cervical-dorsal angle (CDA: angle between upper cervical and lower cervical/upper thoracic vertebra estimated center of mass (COM)), and a -0.5° Rz angle (angle between best fit COM line of lower cervical and upper thoracic vertebral bodies with the vertical).

Treatment protocols

**Figure 3:**
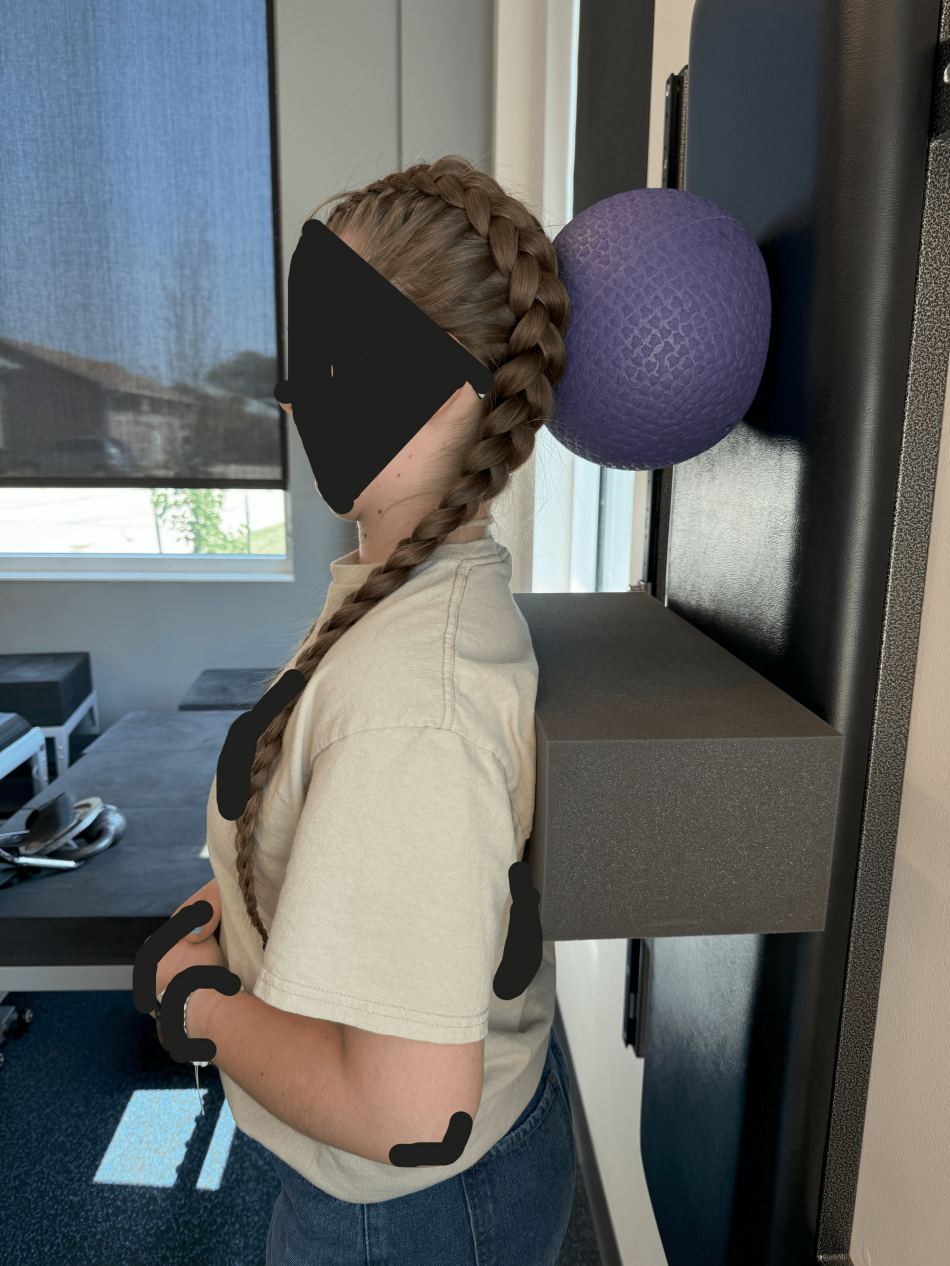
The patient performed posterior head translation and chin-tuck exercises against an elastic ball on the wall to reduce hyperlordosis.

**Figure 4:**
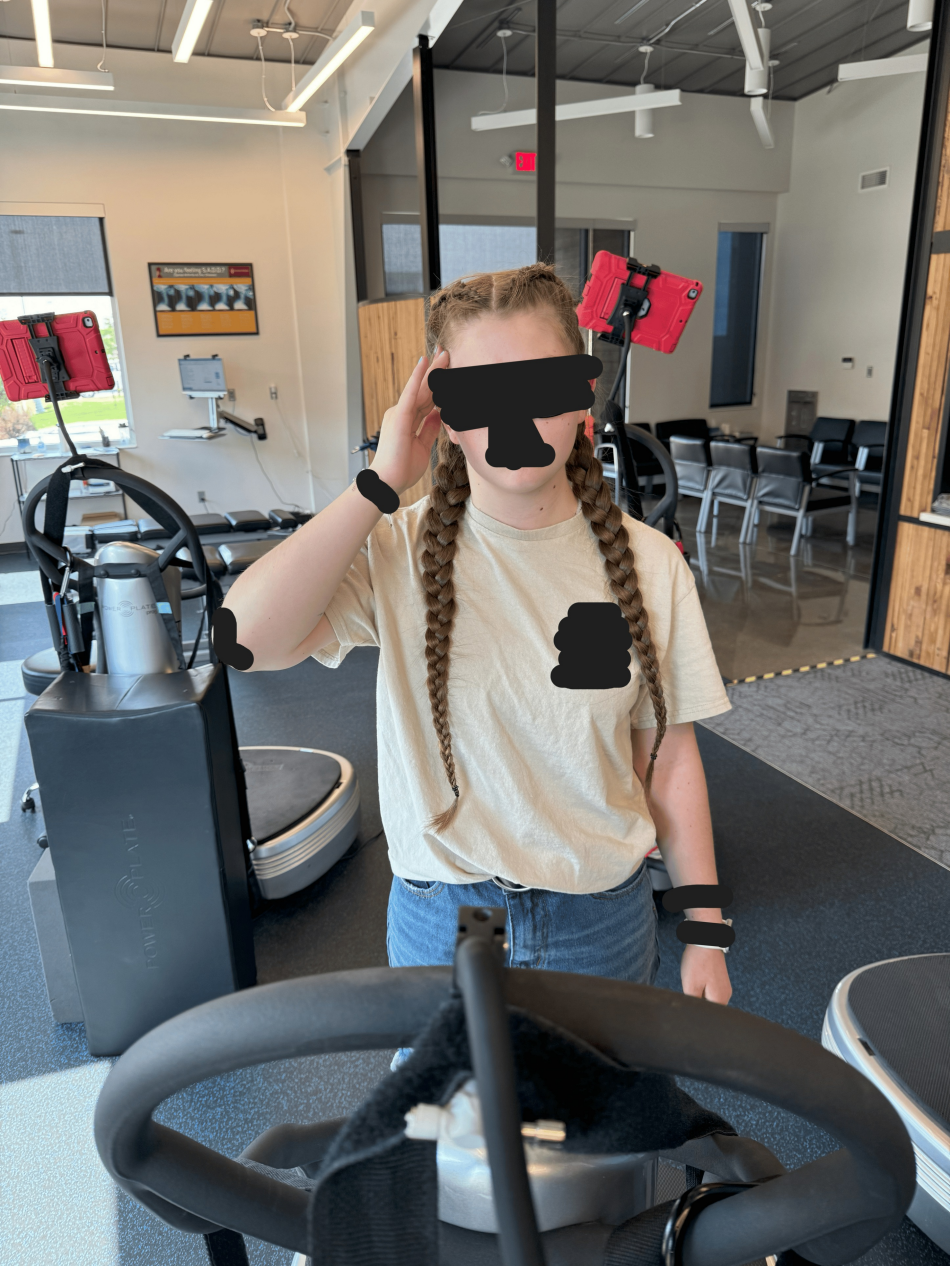
The patient performed strengthening exercises with the head slightly turned right against the resistance of their hand performed on the Power Plate®.

**Figure 5:**
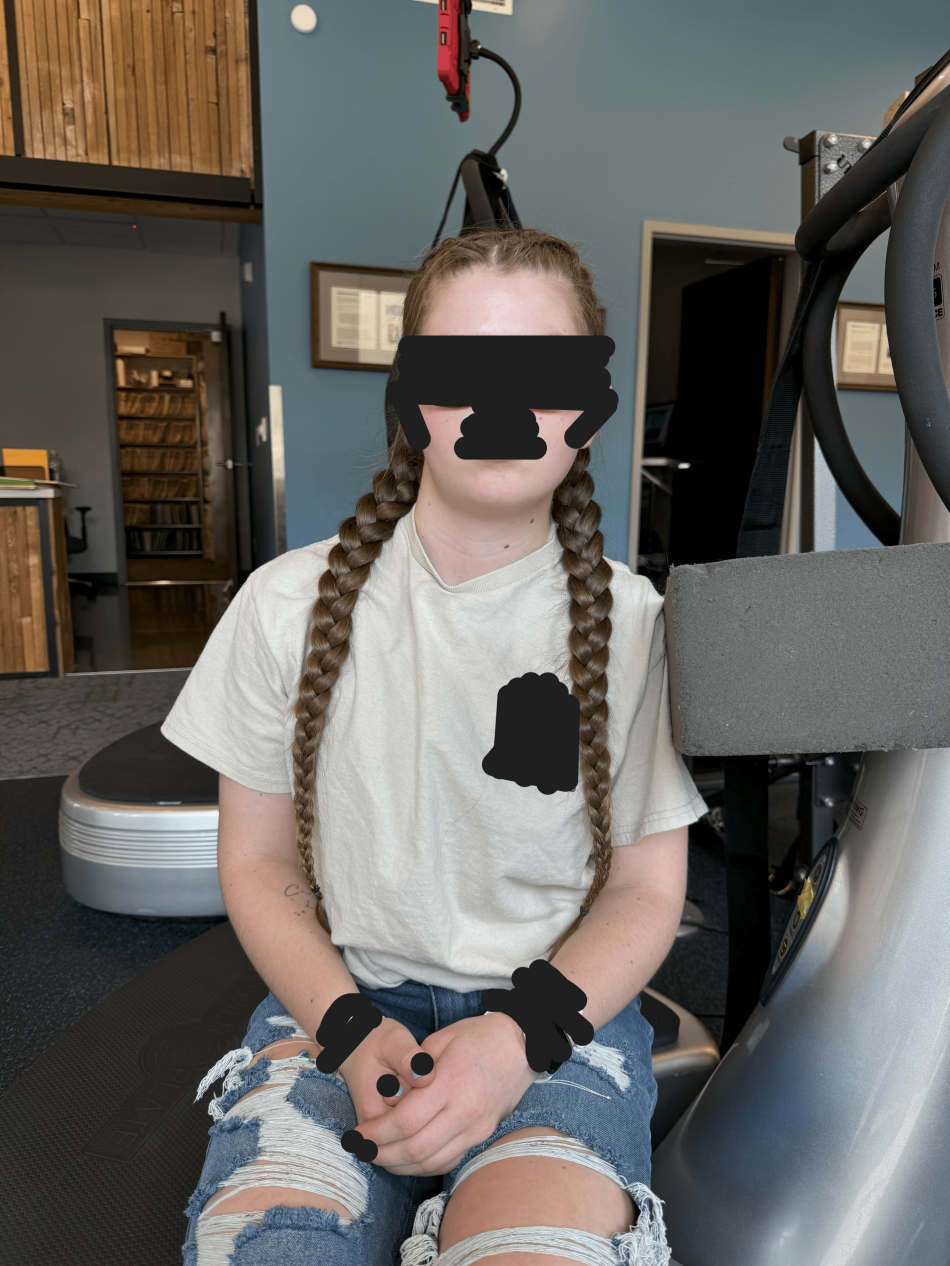
The patient is seated on the Power Plate® with a large block placed on the left shoulder. At eye level, she translates her head in the mirror image of her radiographs to strengthen the muscles and improve the posture toward the midline.

**Figure 6:**
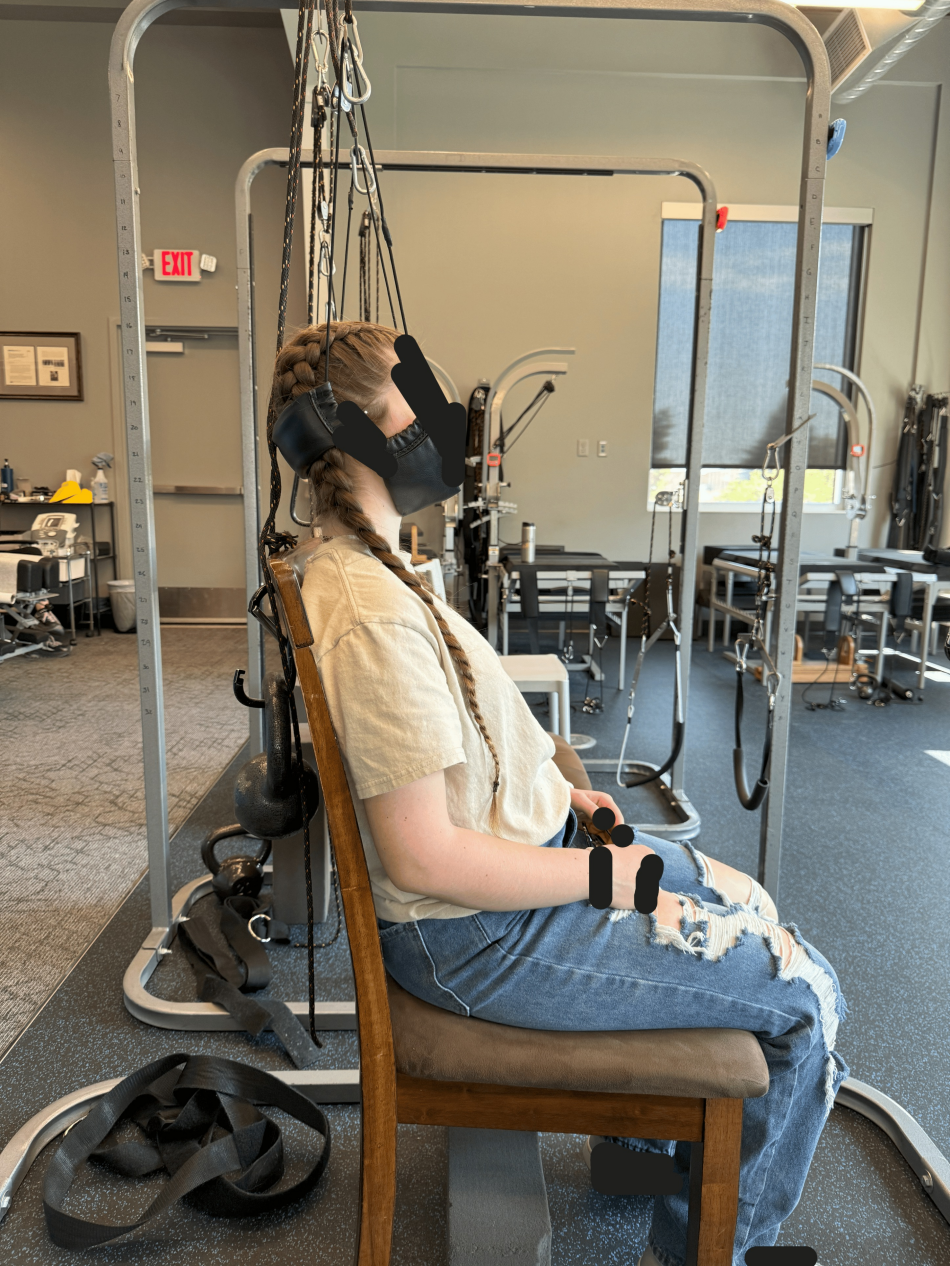
Distraction traction with the patient seated and a weighted harness around the occiput and chin to elongate and flatten the cervical hypolordosis and improve posture.

CBP® technique [15,17-20] was used to improve the cervical spine misalignment. The initial treatment plan was in-office treatments at a frequency of three times per week for four weeks. Mirror image® head retraction exercises with chin tuck pressing the head into a ball placed on the wall were performed for 30 repetitions (Figure 3). Isometric head contractions (cervical rotation and cervical lateral flexions) were held for five seconds for each repetition for one minute for each side and were performed on a Power Plate® (Northbrook, IL, USA) (Figure 4). Left head translations (held for five seconds, rest for two seconds, for two minutes total) were also performed on a Power Plate® (Figure 5). Manual manipulation involving supine mirror image head retractions with a chin tuck was performed on a drop table. Cervical y-axis decompression distraction traction was performed with a weight of 15 lbs for 15 minutes per session (Figure 6). Cryotherapy was applied following treatment for 10 minutes. The patient received 12 treatments over the first 6.5 weeks, then another 12 treatments over another five weeks. Thereafter, treatments were given at a frequency of once per month over a period of a year. Home care involved over-the-door decompression traction using a 15 lb weight at a frequency of 4x/week for the first 12 weeks, and then at a frequency of once per week for the next year. The patient experienced no adverse events and the parents consented to the publication of these results including the radiographs.

Results

Initial Re-evaluation Examination Findings

After 12 in-office treatments over the first 6.5 weeks, an assessment showed some relief and improvements, including a 70% improvement in neck pain and a 10% improvement in headaches, as shown in Table 1. The patient still experienced occasional cervical muscle spasms, palpation demonstrated moderate hypertonicity, point tenderness, and motion restriction in the upper neck mid back and less in the lower back. Cervical and foraminal compression tests were positive with pain localized on the right. Cervical distraction relieved the pain, and the Soto Hall test was positive for pain. Cervical ROM was restricted without pain on flexion and right lateral flexion. All other testing was unremarkable. Cervical radiography showed a 5.3° reduction in lordosis (-38.7° vs. -44.0°), the APL and AHT remained within the standard error of measurement (SEM) at -31.7° (vs. -32.2°) and 8.9 mm (vs. 9.2 mm), respectively. The right head translation was reduced by 12.1 mm (+3.6 mm vs. -8.5 mm), and the CDA and Rz angle were 2.2° and -1.7°, respectively.

Post-treatment Examination Findings

Assessment after 24 in-office treatments over the first 12 weeks showed further improvements, including a 100% improvement in both neck pains and headaches (Table 1). All orthopedic and ROM tests were unremarkable or within normal limits. Static palpation revealed hypertonicity, point tenderness, and motion restriction at C4, T5, and L3. Cervical radiography showed a further decrease in lordosis (-35.8° vs. -38.7°), indicating a total reduction of 8.2°. The APL and AHT measured -29.5° and 9.1 mm, respectively. The coronal head posture translation measured +3.4 mm, and the CDA and Rx angles measured 3.8° and 2.8°, respectively.

Six-Month Follow-Up Assessment Findings

A six-month follow-up assessment after an additional six treatments (30 overall), showed all subjective complaints to be 90-100% improved (Table 1). All orthopedic and ROM tests were normal. The NDI and HDI scored 2% and 0%, respectively. Radiography showed further reduction of the cervical lordosis (-30.3° vs. 35.8°), a total reduction of 13.7°. The APL and AHT measured -27.9 and 9.2 mm, respectively. The head translation regressed slightly (-5.4 mm vs. +3.4 mm), and the CDA and Rz measured 2.8° and -0.6°.

Long-Term Follow-Up Assessment Findings

A 12-month follow-up after approximately 36 in-office treatments demonstrated the patient to be very well (Table 1). All orthopedic and ROM tests were normal. The NDI and HDI scored 8% and 4%, respectively. The radiographic parameters remained consistent, cervical lordosis was -31.4°, APL was -27.9°, and AHT was 11.2 mm. The head translation measured -5.8 mm, CDA was 3.3°, and Rz angle was -0.8°.

## Discussion

This case report featured the reduction in cervical hyperlordosis and improvements in other radiographic parameters accompanied by the relief of neck pain and headaches as well as other symptoms in a 15-year-old female. A one-year follow-up with minimal monthly treatments showed maintenance of postural and symptomatic improvements.

As discussed, most cervical spine subluxation patterns are associated with a loss or straightening (hypolordosis) of the spine [10,11,21,22]. Also, the correction of lordosis, typically increasing lordosis, is associated with the reduction of craniocervical symptoms [21,22]. In the case of cervical hyperlordosis, as featured in the present case, it is logical to presume a correction of lordosis; in this case, a reduction of lordosis would be the ideal treatment plan. Accordingly, the current patient experienced great relief of symptoms corresponding with an approximate 13° reduction and re-establishment of the cervical lordosis toward an age-specific normal alignment.

The cervical curve can be thought of as a three-column system, where Pal et al. found that 36% of the weight loaded through the anterior column formed by the vertebral bodies and discs, and 32% of the loading went through each of the two posterior cervical columns formed by the articular processes [3]. Thus, 64% of the loading occurs through the posterior columns. In the event of cervical hyperlordosis, a greater than normal loading through the posterior columns will occur. Further, in an attempt to accommodate forward gaze [5], the APL will increase as seen in this patient. Surrounding the bony structure are the soft tissues, which, when the cervical spine is subluxated and misaligned, are forced into accommodating such malposition. The static and particularly dynamic stresses and strains exerted onto the soft tissues with normal head movements with a hyperlordotic cervical spine undoubtedly elicit nociceptive tendencies. The reduction of the neck curve toward a more normal alignment could be expected to re-establish biomechanically optimized loading patterns throughout the tissues.

It is suggested that radiographic screening was a key aspect that led to the optimal diagnosis and treatment, and ultimately, the outcome in this adolescent. Without radiographic screening, a biomechanical diagnosis would have been impossible. Although there are some adversaries toward routine radiographic screening of spine patients [27-33], current knowledge supports the fact that spine radiography is the most efficient and safest method to assess spine alignment [34-36].

The limitation of this report is that it is a single case. This case represents the only case, that we are aware of, to show the decrease in lordosis associated with hyperlordosis of the cervical spine. Although cause and effect cannot be validated from a case report, it is presumed that the altered cervical alignment was related to the patient’s symptoms. Future research on treating cervical hyperlordosis is needed as it is essentially non-existent in the scientific literature.

## Conclusions

This case report presents a unique application of the CBP® technique to successfully reduce cervical hyperlordosis and alleviate various craniocervical symptoms in a 15-year-old female. Over the course of a 12-month follow-up, significant improvements were observed not only in spine radiographic parameters but also in the patient's reported neck pain and headaches, demonstrating the potential of CBP® in treating cervical hyperlordosis. In this single case, reducing excessive lordosis led to substantial symptomatic improvement. This report underscores the importance of radiographic screening for accurate diagnosis and biomechanical assessment even in an adolescent/pediatric population. While it remains a single case study, it highlights the need for further research on the treatment of cervical hyperlordosis, which is a rare condition with limited representation in the scientific literature.
